# Cardiometabolic Trajectories Preceding Dementia in Community-Dwelling Older Individuals

**DOI:** 10.1001/jamanetworkopen.2024.58591

**Published:** 2025-02-07

**Authors:** Zimu Wu, Lachlan Cribb, Rory Wolfe, Raj C. Shah, Suzanne G. Orchard, Alice Owen, Robyn L. Woods, Swarna Vishwanath, Trevor T.-J. Chong, Kerry M. Sheets, Anne M. Murray, Joanne Ryan

**Affiliations:** 1 School of Public Health and Preventive Medicine, Monash University, Melbourne, Victoria, Australia; 2 Department of Family and Preventive Medicine, Rush University Medical Center, Chicago, Illinois; 3 Rush Alzheimer’s Disease Center, Rush University Medical Center, Chicago, Illinois; 4 Turner Institute for Brain and Mental Health, School of Psychological Sciences, Monash University, Melbourne, Victoria, Australia; 5 Department of Neurology, Alfred Health, Melbourne, Victoria, Australia; 6 Department of Clinical Neurosciences, St Vincent’s Hospital, Melbourne, Victoria, Australia; 7 Division of Geriatric Medicine, Department of Medicine, Hennepin Healthcare, Minneapolis, Minnesota; 8 Division of Palliative Medicine, Department of Medicine, Hennepin Healthcare, Minneapolis, Minnesota; 9 Berman Center for Outcomes and Clinical Research, Minneapolis, Minnesota

## Abstract

**Importance:**

Poor cardiometabolic health is a risk factor associated with cognitive impairment in later life, but it remains unclear whether cardiometabolic trajectories can serve as early markers associated with dementia.

**Objective:**

To compare cardiometabolic trajectories that precede dementia diagnosis with those among individuals without dementia.

**Design, Setting, and Participants:**

This case-control study analyzed a sample drawn from community-dwelling participants in the Aspirin in Reducing Events in the Elderly (ASPREE) study. Recruitment through primary care physicians occurred between March 2010 and December 2014, with participants followed up for a maximum of 11 years. Dementia cases were matched on sociodemographic characteristics and time of diagnosis to dementia-free controls. Data analysis was performed between February and June 2024.

**Exposures:**

Body mass index (BMI), waist circumference, systolic and diastolic blood pressure, glucose levels, high- and low-density lipoprotein (HDL and LDL) and total cholesterol levels, and triglyceride levels were measured repeatedly between 2010 and 2022.

**Main Outcomes and Measures:**

Dementia (*Diagnostic and Statistical Manual of Mental Disorders* [*Fourth Edition*] criteria) was adjudicated by an international expert panel.

**Results:**

Among 5390 participants (mean [SD] age, 76.9 [4.8] years; 2915 women [54.1%]), there were 2655 individuals (49.3%) with less than 12 years of education. The study included 1078 dementia cases and 4312 controls. Up to a decade before diagnosis, dementia cases compared with controls had lower BMI for all years from −7 years (marginal estimate, 27.52 [95% CI, 27.24 to 27.79] vs 28.00 [95% CI, 27.86 to 28.14]; contrast *P* = 002) to 0 years (marginal estimate, 26.09 [95% CI, 25.81 to 26.36] vs 27.22 [95% CI, 27.09 to 27.36]; contrast *P* < .001) and lower waist circumference for all years from −10 years (marginal estimate, 95.45 cm [95% CI, 94.33 to 96.57 cm] vs 97.35 cm [95% CI, 96.79 to 97.92 cm]; contrast *P* = .003) to 0 years (marginal estimate, 93.90 [95% CI, 93.15 cm to 94.64 cm] vs 96.67 cm [95% CI, 96.30 to 97.05 cm]; contrast *P* < .001); cases also had a faster decline in BMI (linear change β, −0.13 [95% CI, −0.19 to −0.08]) and waist circumference (linear change β, −0.30 cm [95% CI, −0.51 to −0.08 cm]). Compared with controls, cases generally had higher HDL levels, in particular from 5 years (marginal estimate, 62.57 mg/dL [95% CI, 61.59 to 63.56 mg/dL] vs 60.84 mg/dL [95% CI, 60.35 to 61.34 mg/dL]; contrast *P* = .002) to 3 years (marginal estimate, 62.78 mg/dL [95% CI, 61.82 to 63.74 mg/dL] vs 61.08 mg/dL [95% CI, 60.60 to 61.56 mg/dL]; contrast *P* = .002) before dementia but with a decline in levels just before diagnosis (linear change β, −0.47 mg/dL [95% CI, −0.86 to −0.07 mg/dL]). Dementia cases had lower systolic blood pressure and triglyceride levels in the decade before diagnosis and higher LDL and total cholesterol levels, but these were not significantly different from controls.

**Conclusions and Relevance:**

In this study of older individuals, decline in BMI, waist circumference, and HDL occurred up to a decade before dementia diagnosis. These findings provide insights into cardiometabolic changes preceding dementia and the potential for early monitoring and intervention.

## Key Points

**Question:**

What are the trajectories of cardiometabolic risk factors in the 11 years before a dementia diagnosis?

**Findings:**

In this matched case-control study, 1078 dementia cases had a faster decline in body mass index and waist circumference up to 11 years before diagnosis, as well as higher levels of high-density lipoprotein approximately 5 years before diagnosis compared with 4312 controls. Trajectories of blood pressure, triglyceride levels, and glucose levels preceding dementia diagnosis were similar between cases and controls.

**Meaning:**

These findings suggest that weight loss and lipid change may be early indicators associated with underlying cognitive impairments, suggesting the importance of dynamic management of cardiometabolic health.

## Teaser

This case-control study compares trajectories of body mass index, waist circumference, blood pressure, and cholesterol levels over time among individuals with vs without a dementia diagnosis.

## Introduction

Dementia has a long preclinical phase, where neuropathology accumulates years before clinical symptoms and diagnosis.^1^ Early identification of individuals with neuropathology provides opportunities for early interventions and treatments prior to the onset of dementia. There is an association between cardiometabolic function and neurodegeneration,^2^ and late-life cardiometabolic risk factors, including obesity, hypertension, diabetes, and dyslipidemia, are associated with worse brain health and increased dementia risk.^3^ A variety of mechanisms, such as insulin resistance, peripheral inflammation, and oxidative stress, have been proposed to explain this association.^4^ Changes in cardiometabolic factors could thus be indicators of underlying neuropathology, and dynamic monitoring of these factors could provide opportunities for early interventions. However, there are also changes with increasing age in the cardiometabolic system even in the absence of neuropathology; thus, it is important to understand how cardiometabolic patterns that precede dementia diagnosis differ from those that merely reflect natural changes with aging.

A few studies have investigated changes in body mass index (BMI; calculated as weight in kilograms divided by height in meters squared) in individuals who later developed dementia.^5,6,7,8^ However, these studies were limited by small sample sizes and reliance on hospital records for dementia ascertainment. Additionally, some studies focused on select patient groups (eg, individuals with diabetes), limiting the generalizability of the findings. Prior research has explored whether certain cardiometabolic factors, such as blood pressure and glucose and lipid levels, are associated with dementia. However, evidence specifically addressing trajectories that precede a dementia diagnosis remains either inconsistent or lacking.^6,7^ These gaps highlight the need for further research examining a comprehensive spectrum of cardiometabolic factors with regular longitudinal assessment in a well-defined population.

Using data from a large cohort of community-dwelling individuals aged 65 years or older, this study aimed to examine trajectories of cardiometabolic factors in later life. A case-control design was used to compare cardiometabolic trajectories preceding dementia between dementia cases and matched nondementia controls in the 11 prior years.

## Methods

### Participants

Data were obtained from the Aspirin in Reducing Events in the Elderly (ASPREE; 2010-2017) trial and its follow-on observational study, ASPREE Extension (ASPREE-XT; 2019 and onward).^9,10^ Participants aged 65 years and older without major cognitive deficits, physical disability, or cardiovascular disease were recruited in Australia and the US. ASPREE and ASPREE-XT were approved by the ethics review board at each participating institution. The ethics approval and informed consent extend to this study. All participants provided written informed consent. This case-control study is reported following the Strengthening the Reporting of Observational Studies in Epidemiology (STROBE) reporting guideline.

### Assessment of Cardiometabolic Factors

A total of 9 cardiometabolic factors were measured regularly for up to 11 years between 2010 and 2022, following a predefined protocol.^11^ Anthropometric information, including waist circumference (WC), body weight, and height, was obtained using a stadiometer or nonstretch tape and an electronic weighing scale. Systolic and diastolic blood pressure (SBP and DBP) at each visit were the mean of 3 readings taken at 1-minute intervals using an oscillometric monitor. Levels of fasting blood glucose and lipids, including high-density lipoprotein (HDL), low-density lipoprotein (LDL), triglycerides, and total cholesterol, were measured in a local clinic or pathology institution. LDL levels were measured directly when triglyceride levels were too high; otherwise, the Friedwald equation was used to estimate LDL levels.

### Ascertainment of Dementia

Dementia diagnosis was ascertained through a rigorous adjudication process. Trained staff administered the following assessments throughout the study: the Modified Mini-Mental State Examination (3MS, global cognition), Controlled Oral Word Association Test (verbal fluency), Hopkins Verbal Learning Test Revised delayed recall (episodic memory), and psychomotor speed through the Symbol Digit Modalities Test. Suspected dementia cases were identified based on any of these triggering criteria: a score below 78 of 100 or substantial decline (>10.15 points from the 5-year estimated score adjusted by age and education) on the 3MS, self-reported cognitive concern to a general practitioner, a dementia diagnosis in the individual’s medical records, or prescription of cholinesterase inhibitors. Additional assessments were then performed, including the Alzheimer Disease Cooperative Instrumental Study Activities of Daily Living scale, Alzheimer Disease Assessment Scale-Cognitive subscale, Color Trails, Lurian overlapping figures, and self- and partner-reported instrumental activities of daily living. Additional documents, such as medical reports, hospital records, and results from brain imaging and laboratory tests, were collected. An international expert panel of geriatricians and neurologists reviewed all material, and dementia was diagnosed according to *Diagnostic and Statistical Manual of Mental Disorders* (*Fourth Edition*) criteria.^12^

### Nested Case-Control Design

A subsample was selected from the cohort using a nested case-control design, as described previously.^7,8,13^ Participants were excluded if they did not have complete data for all cardiometabolic factors at baseline (968 individuals, including 66 dementia cases) and at least 1 follow-up visit (1415 individuals, including 53 dementia cases). Among the remaining 16 731 participants, 1098 individuals were diagnosed with dementia during follow-up. Each case was matched to 4 controls at the study visit immediately before the time of adjudication (the matching visit). Controls were selected from a pool of participants who were dementia free and not lost to follow-up at the matching visit, without replacement within or between visits. This approach aligned controls with the risk set at the time of dementia diagnosis. Each case was matched with controls by age (older or younger by 1 year), gender, race and ethnicity, and educational level. The ASPREE clinical trial is a binational cohort. Race and ethnicity were self-reported at baseline and assessed to improve the diversity of this cohort, improve the generalizability of results, and reduce health disparities between subpopulations. Categories were Aboriginal or Torres Strait Islander; African American; American Indian; Asian; Hispanic or Latino; Native Hawaiian, Pacific Islander, or Māori; White; more than 1 race; and race or ethnicity could not be determined. A total of 1078 cases were matched with 4312 controls, with 20 cases excluded given that they could not be sufficiently matched (eFigure 1 in Supplement 1).

### Statistical Analysis

Linear mixed-effects models were used to estimate trajectories of each cardiometabolic factor, with random intercepts and slopes for participants and fixed effects for case-control status and covariates. We used a backward timescale across the 12 study visits, with time 0 being the time of the matching visit (time of diagnosis for cases). Participants contributed to the trajectory estimation based on the data available at specific study visits relative to their matching visit. For example, participants matched at the year 11 visit who had baseline data from 11 years previously (baseline) contributed to the estimate at year −11. Variables included in models were case-control status, time, time^2^, and their interactions, as well as matching variables. Differences in the trajectory intercept and slope between cases and controls were examined by the interaction between case-control status and time variables (hereafter, *case-time/case-time^2^*). A Wald test was performed on time/time^2^ for their joint linear and quadratic time effects and their interaction with case-control status. Model-derived marginal estimates of cardiometabolic trajectories were compared at each time (hereafter, *contrast P*).

To test the robustness of results, sensitivity analyses were conducted: excluding individuals who died during follow-up, due to the likely terminal functional decline before death,^14^ and excluding individuals who were diagnosed with dementia or dropped out within the first 5 years after enrollment, to examine the potential bias that could arise from insufficient follow-up time. The threshold of statistical significance was a 2-tailed *P* value <.05. For contrast analysis with multiple testing, statistical significance was adjusted using the Dunn-Šidák correction method.^15^ Analyses were performed using Stata statistical software version 17.0 (StataCorp). Data were analyzed February to June 2024.

## Results

A total of 5390 participants (mean [SD] age at baseline, 76.9 [4.8] years; 2915 women [54.1%]; 4795 Australian White [89.0%], 295 US White [5.5%], and 300 additional groups [5.5%], including 131 African American [2.4%], 92 Hispanic [1.7%], and 77 in any category with <100 participants [1.4%]) were included. Characteristics of participants at enrollment are presented in Table 1.^16^ There were 2655 individuals (49.3%) with less than 12 years of education. The study included 1078 dementia cases (mean [SD] age, 77.1 [4.8] years at baseline; 583 women [54.1%]; 959 Australian White [89.0%], 59 US White [5.5%], and 60 additional groups [5.5%]) and 4312 controls (mean [SD] age, 76.9 [4.8] years at baseline; 2332 women [54.1%]; Australian 3836 White [89.0%], 236 US White [5.5%], and 240 additional groups [5.5%]), who had similar baseline characteristics because of matching. At time 0, the mean [SD] age was 82.1 (5.1) years for cases and 81.9 (5.0) years for controls. Compared with controls, cases were more likely to live alone at home, have dyslipidemia, and be frail. They had a higher proportion of *APOE* ε4 carriers and lower cognitive performance at enrollment.

**Table 1.** Participant Characteristics at Enrollment

Characteristic	Participants, No. (%) (N = 5390)		*P* value
	Cases (n = 1078)	Controls (n = 4312)	
Age at baseline, mean (SD), y			
Continuous	77.1 (4.8)	76.9 (4.8)	.87
65-79	770 (71.4)	3157 (73.2)	.24
≥80	308 (28.6)	1155 (26.8)	
Age at matching visit, mean (SD), y			
Continuous	82.1 (5.1)	81.9 (5.0)	.85
65-79	408 (37.9)	1652 (38.3)	.78
≥80	670 (62.1)	2660 (61.7)	
Gender			
Men	495 (45.9)	1980 (45.9)	>.99
Women	583 (54.1)	2332 (54.1)	
Years of education			
≤11	531 (49.3)	2124 (49.3)	>.99
12-15	306 (28.4)	1224 (28.4)	
≥16	241 (22.3)	964 (22.3)	
Smoking status			
Never	634 (58.8)	2428 (56.3)	.27
Former	419 (38.9)	1762 (40.9)	
Current	25 (2.3)	122 (2.8)	
Alcohol intake			
Never	215 (19.9)	770 (17.9)	.09
Former	64 (5.9)	210 (4.9)	
Current low risk	525 (48.7)	2263 (52.5)	
Current high risk	274 (25.4)	1069 (24.8)	
Living situation			
Living alone at home	400 (37.1)	1432 (33.2)	.02
Living with someone	678 (62.9)	2880 (66.8)	
Hypertension^a^			
Yes	787 (73.0)	3251 (75.4)	.11
No	291 (27.0)	1061 (24.6)	
Diabetes^b^			
Yes	126 (11.7)	445 (10.3)	.19
No	952 (88.3)	3867 (89.7)	
Dyslipidemia^c^			
Yes	745 (69.1)	2827 (65.6)	.03
No	333 (30.9)	1485 (34.4)	
Frailty^d^			
Frail	40 (3.7)	78 (1.8)	<.001
Prefrail	504 (46.8)	1761 (40.8)	
Nonfrail	534 (49.5)	2473 (57.4)	
*APOE* ^e^			
No. with data	816	3484	NA
Yes	355 (43.5)	848 (24.3)	<.001
No	461 (56.5)	2636 (75.7)	
3MS, mean (SD)	90.4 (5.6)	93.5 (4.5)	<.001

Abbreviations: 3MS, Modified Mini-Mental State Examination; NA, not applicable.

^a^ Hypertension was defined as receiving treatment for high blood pressure or having blood pressure greater than 140/90 mm Hg at study entry.

^b^ Diabetes was defined from self-report, a fasting glucose level of 126 mg/dL or greater (≥7 mmol/L), or receiving treatment for diabetes.

^c^ Dyslipidemia was defined as taking cholesterol-lowering medications or having a serum cholesterol level of 212 mg/dL or greater (≥5 mmol/L) in Australia or 240 mg/dL or more (≥6.2 mmol/L) in the US or a low-density lipoprotein level greater than 160 mg/dL (>4.1 mmol/L).

^d^ Frailty was defined based on a frailty index (range, 0-1) constructed by 67 deficits and categorized using cutoff points of greater than 0.21 for frail and from greater than 0.10 to 0.21 for prefrail.^16^

^e^ There are 1090 missing values of *APOE* carrier status in the study sample.

**Figure 1.**
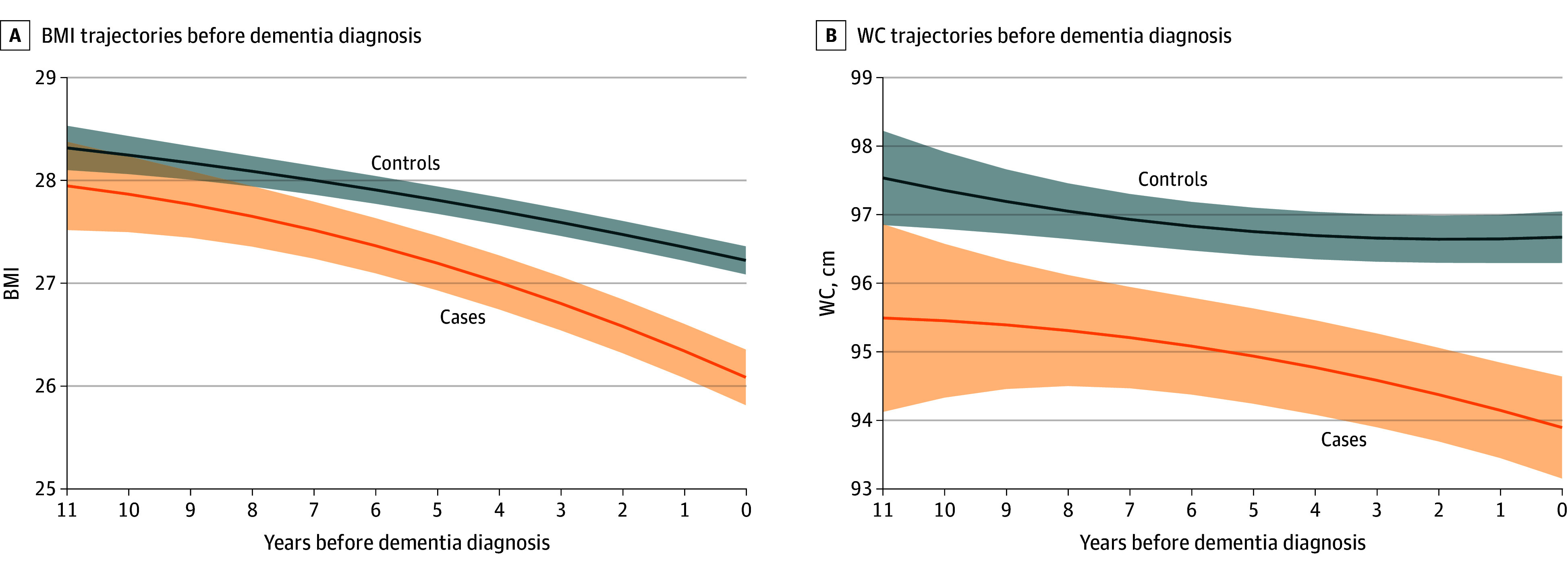
Trajectories of Body Mass Index (BMI) and Waist Circumference (WC) Before Dementia Solid lines and shadings indicate estimated mean trajectories and 95% CIs. Models included case-control status, time, time^2^, and their interaction, as well as age at time 0, gender, race and ethnicity, and years of education. BMI is calculated as weight in kilograms divided by height in meters squared.

For each cardiometabolic factor, trajectories are presented retrospectively from time 0 by case-control status. As shown in Figure 1, declining BMI was observed in cases and controls (eTable 1 in Supplement 1). While controls displayed a steady decline, cases had a lower BMI than controls and a steeper decline beginning at least 11 years before dementia (case-time interaction = −0.13 [95% CI, −0.19 to −0.08]; case-time^2^ interaction = −0.006 [95% CI, −0.012 to 0.001]; *P* < .001) (Table 2). The difference in marginal means between cases and controls was significant from 7 years before diagnosis and became progressively larger over time for all years from −7 years (marginal estimate, 27.52 [95% CI, 27.24 to 27.79] vs 28.00 [95% CI, 27.86 to 28.14]; contrast *P* = .002) to 0 years (marginal estimate, 26.09 [95% CI, 25.81 to 26.36] vs 27.22 [95% CI, 27.09 to 27.36]; contrast *P* < .001) (eTable 2 in Supplement 1). A difference between cases and controls was also observed for WC (Figure 1), with cases having a significantly lower WC 10 years before diagnosis for all years from −10 years (marginal estimate, 95.45 cm [95% CI, 94.33 to 96.57 cm] vs 97.35 cm [95% CI, 96.79 to 97.92 cm]; contrast *P* = .003) to 0 years (marginal estmate, 93.90 cm [95% CI, 93.15 to 94.64 cm] vs 96.67 cm [95% CI, 96.30 to 97.05 cm]; contrast *P* < .001) (eTable 3 in Supplement 1) compared with controls. WC also declined faster for cases compared with controls (case-time interaction = −0.30 cm [95% CI, −0.51 to −0.08 cm]; case-time^2^ interaction = −0.021 cm [95% CI, −0.046 to 0.004 cm]; *P* = .004) (Table 2).

**Table 2.** Differences in Cardiometabolic Trajectories Between 5390 Cases and Controls

Cardiometabolic factor	Coefficient (95% CI)^a^	*P* value^a^
BMI		
Difference in linear change (time)	−0.13 (−0.19 to −0.08)	<.001
Difference in quadratic change (time^2^)	−0.006 (−0.012 to 0.001)	
WC, cm		
Difference in linear change (time)	−0.30 (−0.51 to −0.08)	.004
Difference in quadratic change (time^2^)	−0.021 (−0.046 to 0.004)	
SBP, mm Hg		
Difference in linear change (time)	−0.12 (−0.67 to 0.42)	.68
Difference in quadratic change (time^2^)	−0.02 (−0.09 to 0.04)	
DBP, mm Hg		
Difference in linear change (time)	−0.04 (−0.35 to 0.27)	.49
Difference in quadratic change (time^2^)	−0.01 (−0.05 to 0.02)	
Glucose, mg/dL		
Difference in linear change (time)	0.34 (−0.32 to 1.00)	.18
Difference in quadratic change (time^2^)	0.01 (−0.06 to 0.08)	
HDL, mg/dL		
Difference in linear change (time)	−0.47 (−0.86 to −0.07)	.03
Difference in quadratic change (time^2^)	−0.06 (−0.10 to −0.01)	
LDL, mg/dL		
Difference in linear change (time)	0.78 (−0.31 to 1.87)	.26
Difference in quadratic change (time^2^)	0.10 (−0.02 to 0.23)	
Triglycerides, mg/dL		
Difference in linear change (time)	0.81 (−0.88 to 2.50)	.64
Difference in quadratic change (time^2^)	0.08 (−0.11 to 0.26)	
Total cholesterol, mg/dL		
Difference in linear change (time)	0.21 (−1.04 to 1.46)	.86
Difference in quadratic change (time^2^)	0.03 (−0.10 to 0.17)	

Abbreviations: BMI, body mass index (calculated as weight in kilograms divided by height in meters squared); DBP, diastolic blood pressure; HDL, high-density lipoprotein; LDL, low-density lipoprotein; SBP, systolic blood pressure; WC, waist circumference.

SI conversion factors: To convert glucose to millimoles per liter, multiply by 0.0555; total cholesterol, HDL, and LDL to millimoles per liter, multiply by 0.0259; triglycerides to millimoles per liter, multiply by 0.0113.

^a^ Coefficients and *P* values indicate estimated differences in linear and quadratic time effects between cases and controls (reference). Models included case-control status, time, time^2^, and their interaction, as well as age at time 0, gender, race and ethnicity, and years of education. Joint estimates of time and time^2^ are derived from interaction terms of case-control status with these 2 time variables. A significant *P* value suggests a time effect in time, time^2^, or both.

**Figure 2.**
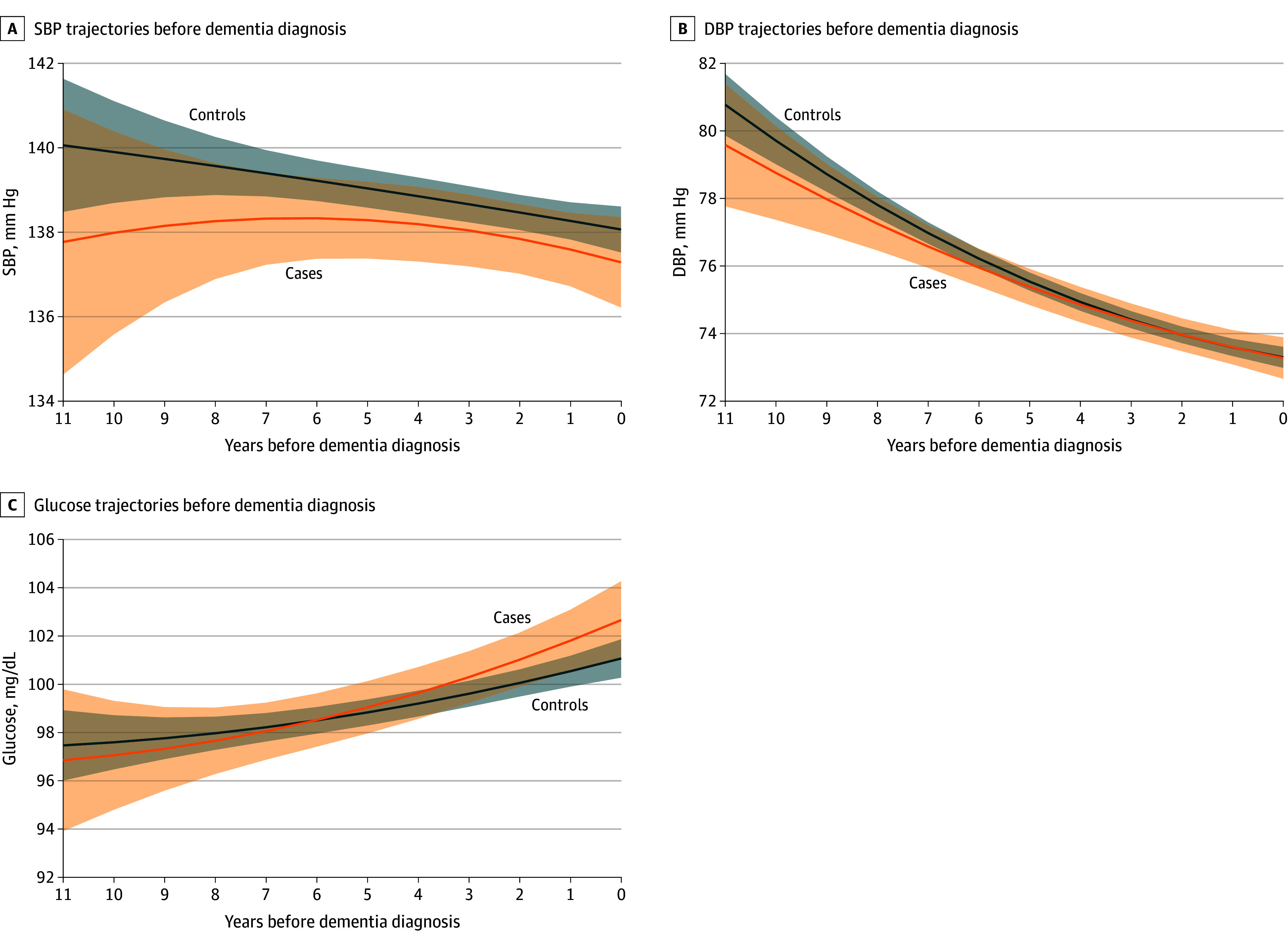
Trajectories of Blood Pressure and Glucose Levels Before Dementia Solid lines and shadings indicate estimated mean trajectories and 95% CIs. Models included case-control status, time, time^2^, and their interaction, as well as age at time 0, gender, race and ethnicity, and years of education. DBP indicates diastolic blood pressure; SBP, systolic blood pressure. SI conversion factor: To convert glucose to millimoles per liter, multiply by 0.0555.

Trajectories of SBP, DBP, and glucose levels are shown in Figure 2. Cases and controls showed a decline in SBP over time, with cases showing lower levels throughout the follow-up, although these differences were not significant (case-time interaction = −0.12 mm Hg [95% CI, −0.67 to 0.42 mm Hg]; case-time^2^ interaction = −0.02 mm Hg [95% CI, −0.09 to 0.04 mm Hg]; *P* = .68) (Table 2; eTable 1 in Supplement 1). DBP declined significantly for cases and controls (eTable 1 in Supplement 1), with similar rates of decline (case-time interaction = −0.04 mm Hg [95% CI, −0.35 to 0.28 mm Hg]; case-time^2^ interaction = −0.01 mm Hg [95% CI, −0.05 to 0.03 mm Hg]; *P* = .49) (Table 2). No significant differences in BP between cases and controls were observed at any time (eTables 4 and 5 in Supplement 1). In contrast, blood glucose levels increased substantially over time in cases and controls (eTable 1 in Supplement 1), without any difference between groups (case-time interaction = 0.34 mg/dL [95% CI, −0.32 to 1.00 mg/dL]; case-time^2^ interaction = 0.01 mg/dL [95% CI, −0.06 to 0.08 mg/dL]; *P* = .18) (Table 2; eTable 6 in Supplement 1). (To convert glucose to millimoles per liter, multiply by 0.0555.)

**Figure 3.**
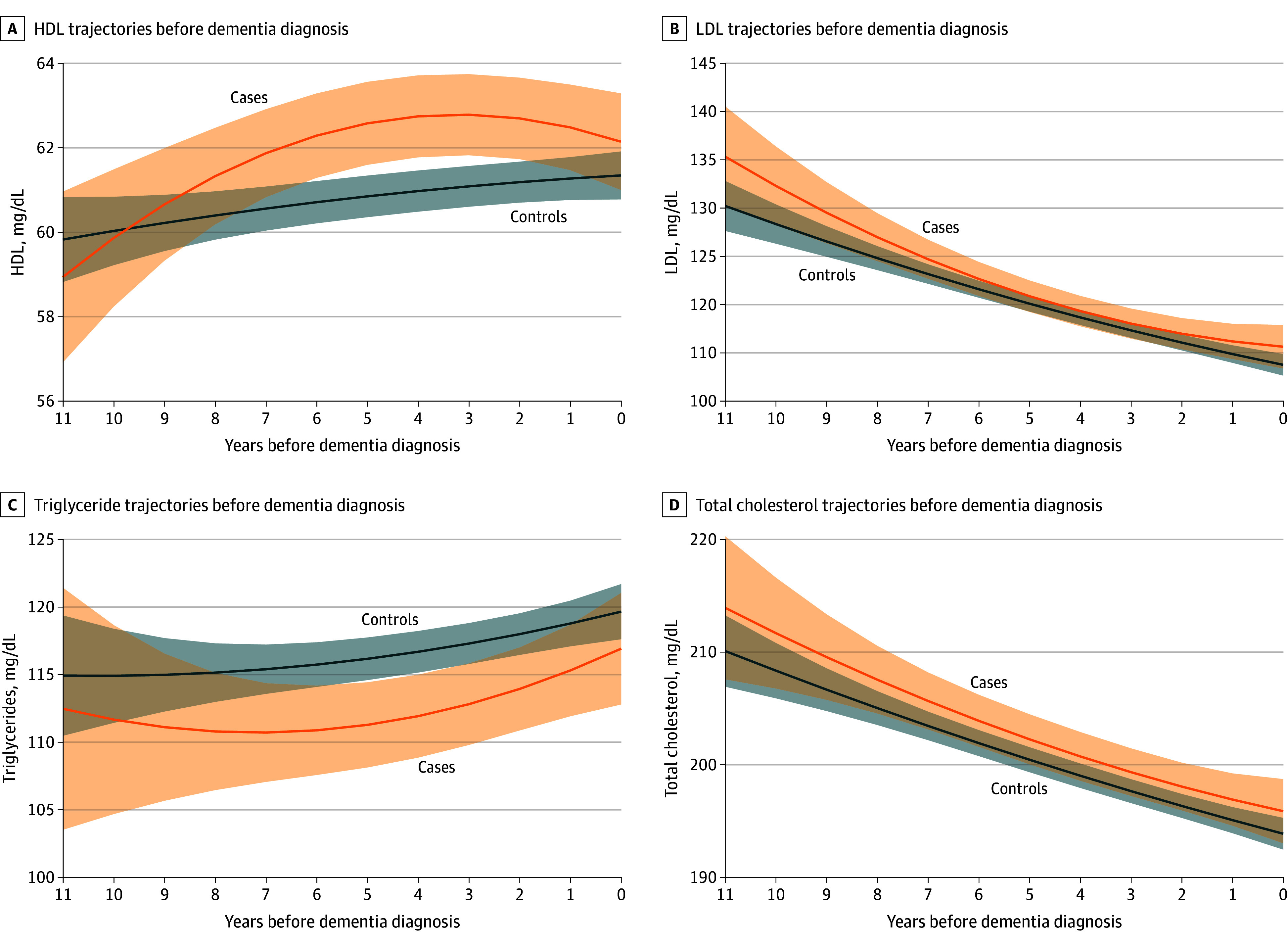
Trajectories of Lipid Levels Before Dementia Solid lines and shadings represent estimated mean trajectories and 95% CIs. Models included case-control status, time, time^2^, and their interaction, as well as age at time 0, gender, race and ethnicity, and years of education. HDL indicates high-density lipoprotein; LDL, low-density lipoprotein. SI conversion factors: To convert total cholesterol, HDL, and LDL to millimoles per liter, multiply by 0.0259; triglycerides to millimoles per liter, multiply by 0.0113.

Figure 3 shows trajectories of blood lipids. A steady increase in HDL across the follow-up was shown in controls (eTable 1 in Supplement 1). However, we observed a greater increase among cases between year −11 and year −4, which then decelerated during the last 4 years leading up to dementia (case-time interaction = −0.47 mg/dL [95% CI, −0.86 to −0.07 mg/dL]; case-time^2^ interaction = −0.06 mg/dL [95% CI, −0.10 to −0.01 mg/dL]; interaction *P* = .03). Cases also showed higher HDL levels than controls 3 to 5 years before diagnosis, while such differences diminished afterward for years −5 (marginal estimate, 62.57 mg/dL [95% CI, 61.59 to 63.56 mg/dL] vs 60.84 mg/dL [95% CI, 60.35 to 61.34 mg/dL]; contrast *P* = .002) to −3 (marginal estimate, 62.78 mg/dL [95% CI, 61.82 to 63.74 mg/dL] vs 61.08 mg/dL [95% CI, 60.60 to 61.56 mg/dL]; contrast *P* = .002) (eTable 7 in Supplement 1). (To convert HDL to millimoles per liter, multiply by 0.0259.) For LDL and total cholesterol levels, we observed a downward trend over the 11 years in cases and controls, with no significant difference in the rate of decline or any marginal mean between cases and controls (Table 2; eTables 8 and 9 in Supplement 1). Triglycerides were consistently higher in controls than cases, with a similar increase over time, but the group difference was not significant (Table 2; eTable 10 in Supplement 1).

Sensitivity analyses excluded individuals who had died over the follow-up or dropped out during the first 5 years after enrollment. The only difference of note was that the rate of change in HDL levels no longer varied by dementia status (eTables 11-12 and eFigures 2-3 in Supplement 1).

## Discussion

In this case-control study among a large cohort of community-dwelling older individuals with a number of cardiometabolic factors assessed regularly over 11 years, we observed a pattern of declining trajectories in BMI, WC, SBP, DBP, and LDL and total cholesterol levels, while blood glucose, HDL, and triglyceride levels generally showed upward trends. Compared with the 4312 controls, the 1078 dementia cases had lower BMI and WC up to 11 years before diagnosis, with a significantly faster decline. Among controls, there was a stable linear increase in HDL levels, which contrasted with the curvilinear patterns in cases that peaked at approximately 3 to 4 years before dementia. Interestingly, the trajectory of blood pressure decline was not significantly different between cases and controls, and LDL and total cholesterol levels were also similar between groups. These results highlight the potential for early identification of individuals at risk of dementia given that body habitus and blood lipid levels in older individuals with prodromal dementia deviate from these measures in the general population up to a decade before the onset of symptoms. Our results also provide insights into the dynamic management of cardiometabolic health and potential windows of opportunity for dementia prevention.

These findings add to and extend previous work that has shown that weight loss is associated with increased dementia risk^6,7,8,17^ and that it may be an early indicator of dementia. The association between body habitus and dementia is thought to be bidirectional. Cognitive decline may occur after weight loss, partially due to homeostatic imbalance.^18^ Meanwhile, underlying cognitive deficits may lead to weight loss via reduced appetite and worsened cooking skills.^19^ Additionally, cases were more likely to live alone than controls in our cohort, potentially having less support for food consumption and preparation. Another explanation is that dementia pathology may affect brain regions that regulate body composition.^20^ To our knowledge, only a few smaller, community-based studies have examined longitudinal trajectories of BMI before dementia,^7,8,21^ and WC has rarely been investigated.^8^ Our findings generally align with those of these studies, although the timing of when these anthropometric measures diverged between cases and controls has been inconsistent. For example, 1 study^7^ with 3925 participants observed divergence 6 years before dementia, with significant differences appearing 1 year before diagnosis. Another study^8^ with 2303 participants found that BMI diverged between cases and controls 8 years before dementia. However, these studies were limited by small case groups (88-785 cases) or significant age differences between cases and controls. Although 1 larger analysis^6^ involved more than 20 000 dementia cases, it exclusively included individuals with diabetes and relied on hospital records for dementia ascertainment. In contrast, the large number of dementia cases and matched controls in our study, combined with the rigorous dementia adjudication process (minimizing misclassification) and with annual assessment of BMI and WC up to 11 years prior to diagnosis, makes this study an important contribution to the field. Our results suggest that weight loss before dementia is likely to occur much earlier than previously reported. Indeed, early neuropathological changes, such as amyloid accumulation, may start up to 15 to 24 years before diagnosis.^1^ Therefore, our results suggest that changes in body habitus may start during the preclinical phase of dementia, preceding symptom manifestation. Theoretically, this may help explain the hypothesis of reverse causation^19,22^ and the obesity paradox in cognitive aging,^23^ which makes excessive body weight appear to be beneficial in later life. More importantly, our results highlight the importance of regular anthropometric assessments with more precise thresholds.

Another innovative finding was that the level of HDL was initially higher in cases, followed by a flattening of the trajectory beginning several years before dementia. Despite the health benefits of HDL,^24^ there have been conflicting results concerning its association with dementia risk.^25,26,27,28^ This may reflect variations in sample characteristics, HDL subspecies, and timing of measurements, suggesting the complexity of HDL functionality. Aligning with our findings, higher baseline HDL was associated with increased dementia risk in our generally healthy cohort,^26^ which is possibly a compensatory response to potential conditions, such as amyloid disposition and delayed lipid metabolism.^29,30^ Reasons for the subsequent decline in HDL at approximately the time of diagnosis are unknown, but reductions in physical activity and food intake as dementia progresses are possible.^31^ This also helps explain the consistently lower triglyceride levels in cases, which may reflect poor diet and compromised nutritional status due to underlying cognitive impairments.^32^ Meanwhile, evidence suggests alternative causal relationships in which specific triglyceride components and medications increasing triglycerides (eg, β-blockers) may promote cognitive function.^33,34^ The absence of significant differences in LDL and glucose trajectories in our study suggests that they may not play a major role during the preclinical phase of dementia. This aligns with evidence indicating that hyperlipidemia and diabetes are risk factors associated with dementia earlier in life.^3^

The declining BP we observed across the entire cohort aligns with prior findings, suggesting cellular changes and functional decline during vascular aging.^6,35,36^ However, we found no significant difference in BP trajectories by case-control status. This is consistent with previous studies showing no association between later-life BP (assessed at 1 time) and incident dementia^37,38^ and aligns with evidence that hypertension is a risk factor associated with dementia only when it persists from midlife.^3,39^ In contrast, some studies have reported that declining BP in later life is associated with higher dementia risk^40^ and a larger number of brain infarcts,^41^ which potentially reflects brain aging processes, such as cerebral atrophy and impaired BP autoregulation.^42^ We observed consistently lower BP in cases than controls, but differences were subtle and did not reach significance despite the relatively large sample size. This may be reflective of a cardiovascular-healthy or robust cohort given that ASPREE included only individuals without cardiovascular disease at recruitment. Moreover, our rigorous process of dementia ascertainment and early capture of cognitive decline through regular assessments likely resulted in an earlier detection of dementia than in other cohort studies, possibly before substantial BP declines occurred.

### Strengths and Limitations

Our study has several strengths. To our knowledge, this is the largest community-based study, with more than 1000 dementia cases, examining precursor cardiometabolic trajectories. The case-control design with longitudinal matching allowed for robust comparability between the 2 groups. The trajectory modeling complemented by contrast analyses at each time visualized longitudinal cardiometabolic changes and highlighted the specific timing of divergence. Moreover, cardiometabolic factors were assessed objectively by trained staff, and all dementia cases were adjudicated rigorously by an expert panel referencing a wide range of clinical evidence. These approaches minimize any bias and error associated with measurements.

This study also has limitations. Participants included in this study were recruited to the ASPREE trial and needed to be willing to participate and without dementia, cardiovascular disease, or substantial physical impairments. This will limit the generalizability of findings. However, participants were recruited via primary care practices, with a balanced gender distribution, a reasonable representation of racial and ethnic minority groups, and diversity of educational attainment and chronic conditions. This suggests that our findings may be broadly relevant to healthy aging populations in similar contexts.

## Conclusions

In this case-control study of community-dwelling older individuals, an accelerated decline in BMI and WC was shown during the decade leading up to dementia. Compared with controls, among whom HDL increased steadily over time, dementia cases initially showed increasingly higher HDL levels, followed by a slight decline over the 4 years before dementia. Additionally, lower BP and triglyceride levels, as well as higher LDL and total cholesterol levels, were shown in cases throughout the follow-up. These findings indicate that certain cardiometabolic factors may deviate from their usual levels before dementia. These differences may be risk factors or early indicators associated with cognitive impairment, suggesting the importance of dynamic monitoring.

## Notes

Supplement 1. **eTable 1.** Time coefficients of cardiometabolic trajectories by cases and controls (N = 5390) **eTable 2.** Means and marginal estimates of BMI at each matching visit by cases and controls (N = 5390) **eTable 3.** Means and marginal estimates of WC at each matching visit by cases and controls (N = 5390) **eTable 4.** Means and marginal estimates of SBP at each matching visit by cases and controls (N = 5390) **eTable 5.** Means and marginal estimates of DBP at each matching visit by cases and controls (N = 5390) **eTable 6.** Means and marginal estimates of glucose levels at each matching visit by cases and controls (N = 5390) **eTable 7.** Means and marginal estimates of HDL levels at each matching visit by cases and controls (N = 5390) **eTable 8.** Means and marginal estimates of LDL levels at each matching visit by cases and controls (N = 5390) **eTable 9.** Means and marginal estimates of total cholesterol levels at each matching visit by cases and controls (N = 5390) **eTable 10.** Means and marginal estimates of triglyceride levels at each matching visit by cases and controls (N = 5390) **eTable 11.** Differences in cardiometabolic trajectories between cases and controls, excluding participants who died during follow-up (n = 4528) **eTable 12.** Differences in cardiometabolic trajectories between cases and controls, excluding participants matched within the first 5 years after enrollment (n = 2965) **eFigure 1.** Flow diagram of participant selection **eFigure 2.** Mean trajectories of cardiometabolic markers before dementia diagnosis by cases (n = 749) and controls (n = 3779), excluding participants who died during follow-up **eFigure 3.** Mean trajectories of cardiometabolic markers before dementia diagnosis by cases (n = 593) and controls (n = 2372), excluding participants matched within the first 5 years after enrollment

Supplement 2.
Data Sharing Statement
