# Supplementary material for: Cardiometabolic Trajectories Preceding Dementia in Community-Dwelling Older Individuals
Source: JAMA Netw Open. 2025 Feb 7;8(2):e2458591. doi: 10.1001/jamanetworkopen.2024.58591 (PMC11806394; doi:10.1001/jamanetworkopen.2024.58591)
Supplement: Supplement 1. — eTable 1. Time coefficients of cardiometabolic trajectories by cases and controls (N = 5390) eTable 2. Means and marginal estimates of BMI at each matching visit by cases and controls (N = 5390) eTable 3. Means and marginal estimates of WC at each matching visit by cases and controls (N = 5390) eTable 4. Means and marginal estimates of SBP at each matching visit by cases and controls (N = 5390) eTable 5. Means and marginal estimates of DBP at each matching visit by cases and controls (N = 5390) eTable 6. Means and marginal estimates of glucose levels at each matching visit by cases and controls (N = 5390) eTable 7. Means and marginal estimates of HDL levels at each matching visit by cases and controls (N = 5390) eTable 8. Means and marginal estimates of LDL levels at each matching visit by cases and controls (N = 5390) eTable 9. Means and marginal estimates of total cholesterol levels at each matching visit by cases and controls (N = 5390) eTable 10. Means and marginal estimates of triglyceride levels at each matching visit by cases and controls (N = 5390) eTable 11. Differences in cardiometabolic trajectories between cases and controls, excluding participants who died during follow-up (n = 4528) eTable 12. Differences in cardiometabolic trajectories between cases and controls, excluding participants matched within the first 5 years after enrollment (n = 2965) eFigure 1. Flow diagram of participant selection eFigure 2. Mean trajectories of cardiometabolic markers before dementia diagnosis by cases (n = 749) and controls (n = 3779), excluding participants who died during follow-up eFigure 3. Mean trajectories of cardiometabolic markers before dementia diagnosis by cases (n = 593) and controls (n = 2372), excluding participants matched within the first 5 years after enrollment [file jamanetwopen-e2458591-s001.pdf]

Supplemental Online Content

Wu Z, Cribb L, Wolfe R, et al. Cardiometabolic trajectories preceding dementia in community-dwelling older individuals. *JAMA Netw Open*. 2025;8(2):e2458591. doi:10.1001/jamanetworkopen.2024.58591

eTable 1. Time coefficients of cardiometabolic trajectories by cases and controls (N = 5390)

eTable 2. Means and marginal estimates of BMI at each matching visit by cases and controls (N = 5390)

eTable 3. Means and marginal estimates of WC at each matching visit by cases and controls (N = 5390)

eTable 4. Means and marginal estimates of SBP at each matching visit by cases and controls (N = 5390)

eTable 5. Means and marginal estimates of DBP at each matching visit by cases and controls (N = 5390)

eTable 6. Means and marginal estimates of glucose levels at each matching visit by cases and controls (N = 5390)

eTable 7. Means and marginal estimates of HDL levels at each matching visit by cases and controls (N = 5390)

eTable 8. Means and marginal estimates of LDL levels at each matching visit by cases and controls (N = 5390)

eTable 9. Means and marginal estimates of total cholesterol levels at each matching visit by cases and controls (N = 5390)

eTable 10. Means and marginal estimates of triglyceride levels at each matching visit by cases and controls (N = 5390)

eTable 11. Differences in cardiometabolic trajectories between cases and controls, excluding participants who died during follow-up (n = 4528)

eTable 12. Differences in cardiometabolic trajectories between cases and controls, excluding participants matched within the first 5 years after enrollment (n = 2965)

eFigure 1. Flow diagram of participant selection

eFigure 2. Mean trajectories of cardiometabolic markers before dementia diagnosis by cases (n = 749) and controls (n = 3779), excluding participants who died during follow-up

eFigure 3. Mean trajectories of cardiometabolic markers before dementia diagnosis by cases (n = 593) and controls (n = 2372), excluding participants matched within the first 5 years after enrollment

This supplemental material has been provided by the authors to give readers additional information about their work.

© 2025 Wu Z et al. *JAMA Network Open*.

Abbreviations:

BMI, body mass index; WC, waist circumference; SBP, systolic blood pressure; DBP, diastolic blood pressure; HDL, high-density lipoprotein; LDL, low-density lipoprotein

eTable 1. Time coefficients of cardiometabolic trajectories by cases and controls (n=5390)

	Cases (n=1078)		Controls (n=4312)		
	**Coefficient (95% CI) [a]**	**P-value [a]**	**Coefficient (95% CI) [a]**	**P-value [a]**	**Interaction p-value [b]**
**BMI**					
Linear change (time)	-0.266 (-0.315 to -0.218)	<0.001	-0.132 (-0.156 to -0.108)	<0.001	<0.001
Quadratic change (time^2)	-0.009 (-0.015 to -0.003)		-0.003 (-0.006 to -0.000)		
**WC**					
Linear change (time)	-0.261 (-0.453 to -0.069)	<0.001	0.036 (-0.061 to 0.133)	0.02	0.004
Quadratic change (time^2)	-0.011 (-0.033 to 0.012)		0.010 (-0.001 to 0.022)		
**SBP**					
Linear change (time)	-0.330 (-0.819 to 0.159)	0.28	-0.206 (-0.452 to 0.040)	<0.001	0.68
Quadratic change (time^2)	-0.026 (-0.085 to 0.033)		-0.002 (-0.032 to 0.027)		
**DBP**					
Linear change (time)	-0.294 (-0.573 to -0.015)	<0.001	-0.256 (-0.396 to -0.116)	<0.001	0.49
Quadratic change (time^2)	0.025 (-0.009 to 0.059)		0.039 (0.021 to 0.056)		
**Glucose**					
Linear change (time)	0.885 (0.293 to 1.477)	<0.001	0.547 (0.258 to 0.835)	<0.001	0.18
Quadratic change (time^2)	0.032 (-0.030 to 0.095)		0.020 (-0.010 to 0.050)		
**HDL**					
Linear change (time)	-0.401 (-0.757 to -0.046)	0.001	0.067 (-0.106 to 0.240)	0.003	0.03
Quadratic change (time^2)	-0.063 (-0.101 to -0.025)		-0.006 (-0.025 to 0.012)		
**LDL**					
Linear change (time)	-0.513 (-1.492 to 0.467)	<0.001	-1.290 (-1.767 to -0.813)	<0.001	0.26
Quadratic change (time^2)	0.149 (0.038 to 0.259)		0.046 (-0.008 to 0.100)		
**Triglycerides**					
Linear change (time)	1.730 (0.206 to 3.254)	0.03	0.921 (0.177 to 1.664)	0.001	0.64
Quadratic change (time^2)	0.121 (-0.048 to 0.289)		0.045 (-0.038 to 0.127)		
**Total cholesterol**					
Linear change (time)	-0.972 (-2.099 to 0.155)	<0.001	-1.181 (-1.731 to -0.631)	<0.001	0.86
Quadratic change (time^2)	0.061 (-0.063 to 0.185)		0.027 (-0.034 to 0.088)		

[a] The coefficients and p-values are derived from linear mixed-effects models that included case-control status, time, time^2, and their interaction, as well as age at time 0, gender, race and ethnicity and years of education. These estimates refer to the within-group rate of change and patterns of acceleration/deceleration in cardiometabolic factors over time.
[b] The interaction p-values are derived from the joint estimates of the interaction between case-control status, and time and time^2. A significant p-value suggests a time effect in either time or time^2, or both.

© 2025 Wu Z et al. *JAMA Network Open*.

eTable 2. Means and marginal estimates of BMI at each matching visit by cases and controls (n=5390)

	Mean (standard deviation)		Marginal estimate (95% CI)		
	Cases (n=1078)	Controls (n=4312)	Cases (n=1078)	Controls (n=4312)	P-value [a]
**Year -11 (n=25)**	24.750 (1.569)	26.849 (2.498)	27.947 (27.517 to 28.377)	28.316 (28.101 to 28.531)	0.13
**Year -10 (n=210)**	26.581 (3.465)	28.056 (4.092)	27.866 (27.497 to 28.235)	28.246 (28.061 to 28.431)	0.07
**Year -9 (n=558)**	27.511 (4.445)	28.139 (4.237)	27.767 (27.443 to 28.091)	28.171 (28.008 to 28.333)	0.03
**Year -8 (n=1077)**	27.353 (4.471)	27.904 (4.316)	27.651 (27.357 to 27.945)	28.089 (27.941 to 28.237)	0.009
**Year -7 (n=1590)**	27.312 (4.505)	27.797 (4.393)	27.517 (27.240 to 27.794)	28.001 (27.862 to 28.140)	0.002 [b]
**Year -6 (n=2216)**	27.107 (4.518)	27.801 (4.376)	27.365 (27.097 to 27.633)	27.908 (27.773 to 28.043)	<0.001 [b]
**Year -5 (n=2874)**	27.042 (4.500)	27.661 (4.326)	27.196 (26.932 to 27.460)	27.808 (27.676 to 27.941)	<0.001 [b]
**Year -4 (n=3579)**	26.811 (4.446)	27.678 (4.419)	27.009 (26.747 to 27.271)	27.703 (27.571 to 27.835)	<0.001 [b]
**Year -3 (n=4003)**	26.625 (4.414)	27.625 (4.417)	26.804 (26.543 to 27.065)	27.592 (27.460 to 27.723)	<0.001 [b]
**Year -2 (n=4370)**	26.646 (4.588)	27.527 (4.402)	26.582 (26.321 to 26.843)	27.475 (27.343 to 27.606)	<0.001 [b]
**Year -1 (n=4335)**	26.294 (4.649)	27.453 (4.440)	26.342 (26.079 to 26.605)	27.351 (27.219 to 27.484)	<0.001 [b]
**Year 0 (n=4111)**	26.366 (4.843)	27.323 (4.494)	26.085 (25.814 to 26.355)	27.222 (27.086 to 27.359)	<0.001 [b]

[a] P-values are based on contrast analyses comparing the marginal estimates of BMI at each time point between cases and controls. This is the post-estimation of linear mixed-effects models that included case-control status, time, time^2, and their interaction, as well as age at time 0, gender, race and ethnicity and years of education.
[b] P-values are statistically significant after adjustment using the Šidák method.

© 2025 Wu Z et al. *JAMA Network Open*.

eTable 3. Means and marginal estimates of WC at each matching visit by cases and controls (n=5390)

	Mean (standard deviation)		Marginal estimate (95% CI)		
	Cases (n=1078)	Controls (n=4312)	Cases (n=1078)	Controls (n=4312)	P-value [a]
**Year -11 (n=25)**	85.400 (12.054)	97.950 (8.281)	95.492 (94.122 to 96.863)	97.535 (96.848 to 98.222)	0.009
**Year -10 (n=210)**	91.667 (9.522)	97.833 (12.179)	95.452 (94.331 to 96.574)	97.353 (96.791 to 97.915)	0.003 [b]
**Year -9 (n=554)**	94.369 (13.029)	97.185 (11.779)	95.391 (94.457 to 96.326)	97.191 (96.723 to 97.659)	<0.001 [b]
**Year -8 (n=1078)**	95.317 (12.688)	96.748 (12.250)	95.309 (94.500 to 96.118)	97.050 (96.645 to 97.456)	<0.001 [b]
**Year -7 (n=1590)**	94.733 (13.126)	96.341 (12.293)	95.206 (94.467 to 95.945)	96.930 (96.560 to 97.300)	<0.001 [b]
**Year -6 (n=2210)**	94.603 (12.619)	96.640 (12.374)	95.082 (94.376 to 95.788)	96.831 (96.476 to 97.185)	<0.001 [b]
**Year -5 (n=2870)**	94.730 (12.902)	96.527 (12.507)	94.937 (94.242 to 95.631)	96.752 (96.403 to 97.101)	<0.001 [b]
**Year -4 (n=3570)**	94.426 (12.656)	96.774 (12.623)	94.770 (94.081 to 95.459)	96.694 (96.348 to 97.040)	<0.001 [b]
**Year -3 (n=3530)**	94.057 (12.405)	96.749 (12.575)	94.583 (93.899 to 95.267)	96.657 (96.313 to 97.001)	<0.001 [b]
**Year -2 (n=3830)**	94.516 (12.999)	96.658 (12.709)	94.375 (93.692 to 95.057)	96.641 (96.297 to 96.984)	<0.001 [b]
**Year -1 (n=3834)**	93.975 (13.255)	96.822 (12.767)	94.145 (93.449 to 94.841)	96.645 (96.294 to 96.996)	<0.001 [b]
**Year 0 (n=3590)**	94.497 (13.752)	96.846 (13.022)	93.895 (93.151 to 94.638)	96.670 (96.295 to 97.046)	<0.001 [b]

[a] P-values are based on contrast analyses comparing the marginal estimates of WC at each time point between cases and controls. This is the post-estimation of linear mixed-effects models that included case-control status, time, time^2, and their interaction, as well as age at time 0, gender, race and ethnicity and years of education.

[b] P-values are statistically significant after adjustment using the Šidák method.

© 2025 Wu Z et al. *JAMA Network Open*.

eTable 4. Means and marginal estimates of SBP at each matching visit by cases and controls (n=5390)

	Mean (standard deviation)		Marginal estimate (95% CI)		
	Cases (n=1078)	Controls (n=4312)	Cases (n=1078)	Controls (n=4312)	P-value [a]
**Year -11 (n=25)**	136.000 (19.287)	136.000 (17.936)	137.771 (134.627 to 140.914)	140.060 (138.484 to 141.637)	0.20
**Year -10 (n=210)**	139.095 (14.531)	137.982 (16.285)	137.987 (135.581 to 140.394)	139.902 (138.695 to 141.109)	0.16
**Year -9 (n=558)**	139.071 (16.106)	138.857 (17.362)	138.152 (136.340 to 139.964)	139.738 (138.830 to 140.647)	0.12
**Year -8 (n=1080)**	137.532 (17.011)	138.731 (16.788)	138.264 (136.893 to 139.635)	139.571 (138.883 to 140.258)	0.09
**Year -7 (n=1595)**	137.684 (17.213)	138.632 (16.520)	138.324 (137.231 to 139.418)	139.398 (138.851 to 139.946)	0.09
**Year -6 (n=2228)**	137.181 (16.301)	139.203 (17.101)	138.333 (137.374 to 139.291)	139.222 (138.742 to 139.701)	0.10
**Year -5 (n=2892)**	137.296 (16.985)	138.715 (16.806)	138.289 (137.379 to 139.199)	139.040 (138.585 to 139.496)	0.15
**Year -4 (n=3589)**	137.655 (17.022)	138.245 (17.008)	138.193 (137.309 to 139.077)	138.855 (138.411 to 139.298)	0.19
**Year -3 (n=4028)**	138.512 (17.436)	138.379 (17.621)	138.045 (137.195 to 138.896)	138.664 (138.237 to 139.092)	0.20
**Year -2 (n=4410)**	137.760 (18.060)	138.487 (17.083)	137.845 (137.022 to 138.669)	138.470 (138.054 to 138.885)	0.18
**Year -1 (n=4394)**	137.502 (17.869)	138.054 (17.383)	137.593 (136.724 to 138.463)	138.270 (137.830 to 138.711)	0.17
**Year 0 (n=4168)**	136.426 (18.348)	137.822 (18.065)	137.289 (136.218 to 138.361)	138.067 (137.523 to 138.610)	0.20

[a] P-values are based on contrast analyses comparing the marginal estimates of SBP at each time point between cases and controls. This is the post-estimation of linear mixed-effects models that included case-control status, time, time^2, and their interaction, as well as age at time 0, gender, race and ethnicity and years of education.

Note: No p-value is statistically significant after adjustment.

© 2025 Wu Z et al. *JAMA Network Open*.

eTable 5. Means and marginal estimates of DBP at each matching visit by cases and controls (n=5390)

	Mean (standard deviation)		Marginal estimate (95% CI)		
	Cases (n=1078)	Controls (n=4312)	Cases (n=1078)	Controls (n=4312)	P-value [a]
**Year -11 (n=25)**	75.000 (7.000)	74.900 (9.867)	79.581 (77.768 to 81.394)	80.772 (79.863 to 81.681)	0.25
**Year -10 (n=210)**	78.000 (9.090)	78.667 (9.846)	78.754 (77.367 to 80.141)	79.707 (79.012 to 80.403)	0.23
**Year -9 (n=558)**	78.009 (9.317)	77.603 (10.182)	77.977 (76.934 to 79.021)	78.719 (78.196 to 79.243)	0.21
**Year -8 (n=1080)**	76.271 (10.616)	76.597 (9.919)	77.252 (76.461 to 78.042)	77.809 (77.413 to 78.205)	0.22
**Year -7 (n=1595)**	75.350 (10.113)	75.510 (9.794)	76.577 (75.944 to 77.209)	76.975 (76.658 to 77.292)	0.27
**Year -6 (n=2228)**	74.098 (9.977)	75.431 (10.045)	75.953 (75.394 to 76.511)	76.218 (75.939 to 76.498)	0.40
**Year -5 (n=2892)**	75.059 (9.806)	75.060 (9.990)	75.379 (74.845 to 75.914)	75.539 (75.271 to 75.806)	0.60
**Year -4 (n=3589)**	74.131 (10.064)	74.702 (10.146)	74.857 (74.335 to 75.379)	74.936 (74.674 to 75.198)	0.79
**Year -3 (n=4028)**	74.526 (10.271)	74.548 (10.244)	74.385 (73.882 to 74.889)	74.411 (74.157 to 74.664)	0.93
**Year -2 (n=4411)**	74.149 (10.288)	74.393 (10.154)	73.965 (73.479 to 74.451)	73.962 (73.717 to 74.207)	0.99
**Year -1 (n=4394)**	73.721 (10.325)	73.891 (10.045)	73.595 (73.088 to 74.101)	73.590 (73.334 to 73.847)	0.99
**Year 0 (n=4168)**	73.378 (10.379)	73.639 (10.365)	73.275 (72.662 to 73.889)	73.296 (72.985 to 73.607)	0.95

[a] P-values are based on contrast analyses comparing the marginal estimates of DBP at each time point between cases and controls. This is the post-estimation of linear mixed-effects models that included case-control status, time, time^2, and their interaction, as well as age at time 0, gender, race and ethnicity and years of education.

Note: No p-value is statistically significant after adjustment.

© 2025 Wu Z et al. *JAMA Network Open*.

eTable 6. Means and marginal estimates of glucose at each matching visit by cases and controls (n=5390)

	Mean (standard deviation)		Marginal estimate (95% CI)		
	**Cases (n=1078)**	**Controls (n=4312)**	**Cases (n=1078)**	**Controls (n=4312)**	**P-value [a]**
**Year -11 (n=25)**	99.000 (18.532)	98.370 (23.398)	96.854 (93.917 to 99.791)	97.469 (96.011 to 98.926)	0.71
**Year -10 (n=210)**	102.010 (25.162)	97.751 (16.057)	97.057 (94.803 to 99.312)	97.597 (96.475 to 98.718)	0.67
**Year -9 (n=536)**	96.131 (12.551)	99.731 (23.645)	97.326 (95.595 to 99.056)	97.764 (96.901 to 98.628)	0.66
**Year -8 (n=1027)**	98.512 (15.840)	99.105 (16.715)	97.659 (96.284 to 99.034)	97.972 (97.285 to 98.659)	0.69
**Year -7 (n=1522)**	99.565 (20.366)	99.315 (17.923)	98.057 (96.876 to 99.238)	98.220 (97.630 to 98.810)	0.81
**Year -6 (n=2093)**	99.133 (17.333)	99.544 (18.731)	98.520 (97.416 to 99.624)	98.507 (97.956 to 99.058)	0.98
**Year -5 (n=2666)**	99.927 (18.929)	99.369 (18.850)	99.048 (97.966 to 100.130)	98.835 (98.294 to 99.375)	0.73
**Year -4 (n=3259)**	100.239 (21.101)	99.427 (18.483)	99.641 (98.567 to 100.714)	99.202 (98.665 to 99.739)	0.47
**Year -3 (n=3578)**	100.435 (25.728)	99.663 (19.648)	100.299 (99.225 to 101.372)	99.609 (99.071 to 100.147)	0.26
**Year -2 (n=3580)**	100.196 (21.017)	99.998 (21.030)	101.021 (99.899 to 102.144)	100.056 (99.495 to 100.618)	0.13
**Year -1 (n=3260)**	101.075 (23.342)	100.125 (20.693)	101.809 (100.525 to 103.093)	100.543 (99.904 to 101.183)	0.08
**Year 0 (n=2795)**	99.968 (24.286)	99.754 (22.101)	102.661 (101.052 to 104.271)	101.070 (100.275 to 101.866)	0.08

[a] P-values are based on contrast analyses comparing the marginal estimates of glucose at each time point between cases and controls. This is the post-estimation of linear mixed-effects models that included case-control status, time, $time^2$, and their interaction, as well as age at time 0, gender, race and ethnicity and years of education.

Note: No p-value is statistically significant after adjustment.

© 2025 Wu Z et al. *JAMA Network Open*.

eTable 7. Means and marginal estimates of HDL at each matching visit by cases and controls (n=5390)

	Mean (standard deviation)		Marginal estimate (95% CI)		
	**Cases (n=1078)**	**Controls (n=4312)**	**Cases (n=1078)**	**Controls (n=4312)**	**P-value [a]**
**Year -11 (n=25)**	58.000 (9.874)	54.135 (14.628)	58.935 (56.915 to 60.956)	59.823 (58.817 to 60.829)	0.44
**Year -10 (n=210)**	61.964 (17.684)	61.735 (17.920)	59.856 (58.232 to 61.481)	60.026 (59.216 to 60.836)	0.86
**Year -9 (n=540)**	62.276 (16.278)	60.893 (16.187)	60.652 (59.320 to 61.984)	60.215 (59.550 to 60.880)	0.57
**Year -8 (n=1033)**	61.705 (16.640)	60.198 (16.443)	61.321 (60.178 to 62.464)	60.392 (59.820 to 60.963)	0.15
**Year -7 (n=1515)**	61.325 (17.571)	60.400 (16.828)	61.864 (60.823 to 62.906)	60.556 (60.035 to 61.076)	0.03
**Year -6 (n=2109)**	62.605 (18.498)	60.521 (17.083)	62.282 (61.283 to 63.280)	60.706 (60.207 to 61.206)	0.006
**Year -5 (n=2681)**	63.005 (18.103)	61.237 (17.459)	62.573 (61.591 to 63.555)	60.844 (60.353 to 61.336)	0.002 [b]
**Year -4 (n=3272)**	62.383 (18.453)	61.073 (17.343)	62.738 (61.768 to 63.708)	60.969 (60.482 to 61.456)	0.001 [b]
**Year -3 (n=3149)**	63.408 (19.444)	60.983 (17.698)	62.778 (61.819 to 63.737)	61.081 (60.599 to 61.564)	0.002 [b]
**Year -2 (n=3101)**	62.796 (18.896)	60.935 (17.654)	62.691 (61.728 to 63.654)	61.181 (60.697 to 61.665)	0.006
**Year -1 (n=2814)**	61.330 (18.305)	61.008 (17.693)	62.479 (61.467 to 63.491)	61.267 (60.761 to 61.773)	0.04
**Year 0 (n=2318)**	61.088 (18.235)	60.885 (17.978)	62.140 (60.997 to 63.283)	61.340 (60.773 to 61.908)	0.22

[a] P-values are based on contrast analyses comparing the marginal estimates of HDL at each time point between cases and controls. This is the post-estimation of linear mixed-effects models that included case-control status, time, time^2, and their interaction, as well as age at time 0, gender, race and ethnicity and years of education.

[b] P-values are statistically significant after adjustment using the Šidák method.

© 2025 Wu Z et al. *JAMA Network Open*.

eTable 8. Means and marginal estimates of LDL at each matching visit by cases and controls (n=5390)

	Mean (standard deviation)		Marginal estimate (95% CI)		
	**Cases (n=1078)**	**Controls (n=4312)**	**Cases (n=1078)**	**Controls (n=4312)**	**P-value [a]**
**Year -11 (n=25)**	129.140 (37.671)	122.190 (25.970)	134.386 (128.144 to 140.628)	128.262 (125.153 to 131.370)	0.09
**Year -10 (n=210)**	116.457 (29.031)	119.796 (33.486)	130.752 (125.860 to 135.643)	126.007 (123.567 to 128.446)	0.09
**Year -9 (n=540)**	120.871 (33.889)	120.155 (31.302)	127.414 (123.614 to 131.214)	123.843 (121.946 to 125.740)	0.10
**Year -8 (n=1033)**	120.553 (35.650)	117.862 (33.032)	124.374 (121.396 to 127.353)	121.772 (120.284 to 123.260)	0.13
**Year -7 (n=1512)**	117.140 (36.259)	116.404 (34.278)	121.631 (119.205 to 124.058)	119.793 (118.580 to 121.005)	0.18
**Year -6 (n=2108)**	114.143 (35.702)	115.151 (34.157)	119.186 (117.075 to 121.298)	117.905 (116.851 to 118.960)	0.29
**Year -5 (n=2679)**	114.992 (36.207)	114.064 (33.954)	117.038 (115.081 to 118.996)	116.110 (115.132 to 117.088)	0.41
**Year -4 (n=3270)**	114.739 (35.138)	113.223 (34.126)	115.188 (113.300 to 117.075)	114.406 (113.461 to 115.351)	0.47
**Year -3 (n=3142)**	113.235 (34.231)	112.842 (34.912)	113.634 (111.766 to 115.502)	112.794 (111.858 to 113.731)	0.43
**Year -2 (n=3097)**	114.358 (35.050)	112.574 (34.442)	112.378 (110.441 to 114.316)	111.275 (110.304 to 112.245)	0.32
**Year -1 (n=2814)**	113.495 (35.993)	110.964 (33.951)	111.420 (109.232 to 113.608)	109.847 (108.757 to 110.936)	0.21
**Year 0 (n=2313)**	113.301 (36.510)	112.120 (34.921)	110.759 (108.055 to 113.462)	108.510 (107.176 to 109.845)	0.14

[a] P-values are based on contrast analyses comparing the marginal estimates of LDL at each time point between cases and controls. This is the post-estimation of linear mixed-effects models that included case-control status, time, time^2, and their interaction, as well as age at time 0, gender, race and ethnicity and years of education.

Note: No p-value is statistically significant after adjustment.

© 2025 Wu Z et al. *JAMA Network Open*.

eTable 9. Means and marginal estimates of total cholesterol at each matching visit by cases and controls (n=5390)

	Mean (standard deviation)		Marginal estimate (95 CI)		
	**Cases (n=1078)**	**Controls (n=4312)**	**Cases (n=1078)**	**Controls (n=4312)**	**P-value [a]**
**Year -11 (n=25)**	209.600 (37.441)	203.210 (36.741)	213.936 (207.582 to 220.291)	210.096 (206.929 to 213.264)	0.29
**Year -10 (n=210)**	201.662 (32.109)	205.895 (38.209)	211.685 (206.765 to 216.605)	208.354 (205.897 to 210.810)	0.23
**Year -9 (n=543)**	205.076 (38.762)	204.825 (34.961)	209.555 (205.759 to 213.352)	206.665 (204.767 to 208.563)	0.18
**Year -8 (n=1036)**	205.343 (39.983)	201.965 (36.163)	207.547 (204.544 to 210.551)	205.029 (203.527 to 206.531)	0.14
**Year -7 (n=1531)**	201.878 (40.323)	200.701 (38.270)	205.661 (203.129 to 208.194)	203.447 (202.181 to 204.712)	0.12
**Year -6 (n=2128)**	199.502 (40.379)	199.525 (38.320)	203.897 (201.586 to 206.209)	201.918 (200.764 to 203.072)	0.13
**Year -5 (n=2703)**	200.797 (40.780)	198.682 (38.122)	202.255 (200.033 to 204.477)	200.443 (199.333 to 201.553)	0.15
**Year -4 (n=3311)**	199.415 (39.930)	197.955 (38.721)	200.735 (198.569 to 202.900)	199.021 (197.937 to 200.105)	0.17
**Year -3 (n=3184)**	199.796 (40.343)	197.653 (39.260)	199.336 (197.233 to 201.450)	197.653 (196.592 to 198.713)	0.16
**Year -2 (n=3146)**	199.854 (39.678)	197.259 (38.832)	198.060 (195.939 to 200.181)	196.338 (195.274 to 197.402)	0.15
**Year -1 (n=2858)**	196.905 (38.908)	195.230 (38.322)	196.905 (194.583 to 199.227)	195.077 (193.919 to 196.235)	0.17
**Year 0 (n=2375)**	196.961 (40.490)	196.565 (39.721)	195.872 (193.023 to 198.722)	193.869 (192.462 to 195.277)	0.21

[a] P-values are based on contrast analyses comparing the marginal estimates of total cholesterol at each time point between cases and controls. This is the post-estimation of linear mixed-effects models that included case-control status, time, time^2, and their interaction, as well as age at time 0, gender, race and ethnicity and years of education.

Note: No p-value is statistically significant after adjustment.

© 2025 Wu Z et al. *JAMA Network Open*.

eTable 10. Means and marginal estimates of triglycerides at each matching visit by cases and controls (n=5390)

	Mean (standard deviation)		Marginal estimate (95% CI)		
	**Cases (n=1078)**	**Controls (n=4312)**	**Cases (n=1078)**	**Controls (n=4312)**	**P-value [a]**
**Year -11 (n=25)**	113.340 (29.637)	133.745 (62.430)	112.469 (103.526 to 121.413)	114.926 (110.479 to 119.373)	0.63
**Year -10 (n=210)**	115.055 (67.487)	121.929 (60.210)	111.669 (104.685 to 118.652)	114.911 (111.433 to 118.388)	0.42
**Year -9 (n=543)**	109.795 (53.473)	120.095 (60.290)	111.110 (105.669 to 116.550)	114.985 (112.272 to 117.698)	0.21
**Year -8 (n=1036)**	113.838 (56.435)	118.272 (56.709)	110.791 (106.458 to 115.124)	115.148 (112.984 to 117.311)	0.08
**Year -7 (n=1530)**	114.810 (52.699)	118.434 (56.170)	110.714 (107.064 to 114.364)	115.400 (113.577 to 117.223)	0.02
**Year -6 (n=2124)**	111.373 (52.039)	117.842 (55.084)	110.878 (107.574 to 114.181)	115.741 (114.091 to 117.392)	0.01
**Year -5 (n=2701)**	113.815 (50.376)	116.664 (54.391)	111.282 (108.131 to 114.433)	116.172 (114.596 to 117.747)	0.007
**Year -4 (n=3304)**	111.429 (51.715)	116.609 (52.968)	111.928 (108.860 to 114.996)	116.691 (115.154 to 118.228)	0.007
**Year -3 (n=3180)**	114.129 (57.041)	117.873 (56.230)	112.815 (109.797 to 115.832)	117.300 (115.786 to 118.814)	0.009
**Year -2 (n=3140)**	113.131 (53.737)	118.460 (57.263)	113.942 (110.872 to 117.013)	117.998 (116.458 to 119.537)	0.02
**Year -1 (n=2845)**	114.716 (62.750)	116.762 (54.776)	115.311 (111.923 to 118.700)	118.785 (117.095 to 120.474)	0.07
**Year 0 (n=2370)**	113.273 (58.052)	119.428 (57.055)	116.921 (112.792 to 121.050)	119.661 (117.619 to 121.702)	0.24

[a] P-values are based on contrast analyses comparing the marginal estimates of triglycerides at each time point between cases and controls. This is the post-estimation of linear mixed-effects models that included case-control status, time, time^2, and their interaction, as well as age at time 0, gender, race and ethnicity and years of education.

Note: No p-value is statistically significant after adjustment.

© 2025 Wu Z et al. *JAMA Network Open*.

eTable 11. Differences in cardiometabolic trajectories between cases and controls, excluding participants who died during follow-up (n=4528)

	Coefficient (95% CI) [a]	P-value [a]
**BMI**		
Difference in linear change (time)	-0.151 (-0.210 to -0.092)	<0.001
Difference in quadratic change (time^2)	-0.006 (-0.013 to 0.000)	
**WC**		
Difference in linear change (time)	-0.321 (-0.558 to -0.085)	0.01
Difference in quadratic change (time^2)	-0.025 (-0.052 to 0.002)	
**SBP**		
Difference in linear change (time)	0.059 (-0.542 to 0.660)	0.61
Difference in quadratic change (time^2)	-0.007 (-0.077 to 0.063)	
**DBP**		
Difference in linear change (time)	0.077 (-0.270 to 0.424)	0.38
Difference in quadratic change (time^2)	-0.002 (-0.043 to 0.039)	
**Glucose**		
Difference in linear change (time)	0.422 (-0.337 to 1.180)	0.12
Difference in quadratic change (time^2)	0.015 (-0.061 to 0.091)	
**HDL**		
Difference in linear change (time)	-0.175 (-0.639 to 0.289)	0.37
Difference in quadratic change (time^2)	-0.028 (-0.075 to 0.020)	
**LDL**		
Difference in linear change (time)	1.106 (-0.258 to 2.470)	0.19
Difference in quadratic change (time^2)	0.130 (-0.010 to 0.270)	
**Triglycerides**		
Difference in linear change (time)	0.507 (-1.531 to 2.545)	0.79
Difference in quadratic change (time^2)	0.029 (-0.183 to 0.240)	
**Total cholesterol**		
Difference in linear change (time)	0.476 (-0.958 to 1.909)	0.65
Difference in quadratic change (time^2)	0.067 (-0.085 to 0.219)	

[a] The coefficients and p-values indicate the estimated difference in cardiometabolic trajectories (intercept and slope) between cases and controls (reference). The models included case-control status, time, time^2, and their interaction, as well as age at time 0, gender, race and ethnicity and years of education. The joint estimates of time and time^2 are derived from the interaction terms of case-control status with these two time variables. A significant p-value suggests a time effect in either time or time^2, or both.

© 2025 Wu Z et al. *JAMA Network Open*.

eTable 12. Differences in cardiometabolic trajectories between cases and controls, excluding participants matched within the first five years after enrollment (n=2965)

	Coefficient (95% CI) [a]	P-value [a]
**BMI**		
Difference in linear change (time)	-0.104 (-0.174 to -0.034)	<0.001
Difference in quadratic change ($time^2$)	-0.004 (-0.011 to 0.004)	
**WC**		
Difference in linear change (time)	-0.143 (-0.412 to 0.127)	0.31
Difference in quadratic change ($time^2$)	-0.008 (-0.038 to 0.022)	
**SBP**		
Difference in linear change (time)	0.021 (-0.653 to 0.695)	0.89
Difference in quadratic change ($time^2$)	-0.005 (-0.081 to 0.071)	
**DBP**		
Difference in linear change (time)	-0.093 (-0.473 to 0.287)	0.72
Difference in quadratic change ($time^2$)	-0.015 (-0.059 to 0.028)	
**Glucose**		
Difference in linear change (time)	0.356 (-0.600 to 1.312)	0.30
Difference in quadratic change ($time^2$)	0.012 (-0.078 to 0.102)	
**HDL**		
Difference in linear change (time)	-0.149 (-0.721 to 0.424)	0.22
Difference in quadratic change ($time^2$)	-0.029 (-0.084 to 0.026)	
**LDL**		
Difference in linear change (time)	0.351 (-1.312 to 2.013)	0.50
Difference in quadratic change ($time^2$)	0.063 (-0.097 to 0.223)	
**Triglycerides**		
Difference in linear change (time)	2.799 (0.428 to 5.171)	0.06
Difference in quadratic change ($time^2$)	0.236 (0.001 to 0.471)	
**Total cholesterol**		
Difference in linear change (time)	0.082 (-1.675 to 1.839)	0.82
Difference in quadratic change ($time^2$)	0.028 (-0.146 to 0.202)	

[a] The coefficients and p-values indicate the estimated difference in cardiometabolic trajectories (intercept and slope) between cases and controls (reference). The models included case-control status, time, $time^2$, and their interaction, as well as age at time 0, gender, race and ethnicity and years of education. The joint estimates of time and $time^2$ are derived from the interaction terms of case-control status with these two time variables. A significant p-value suggests a time effect in either time or $time^2$, or both.

© 2025 Wu Z et al. *JAMA Network Open*.

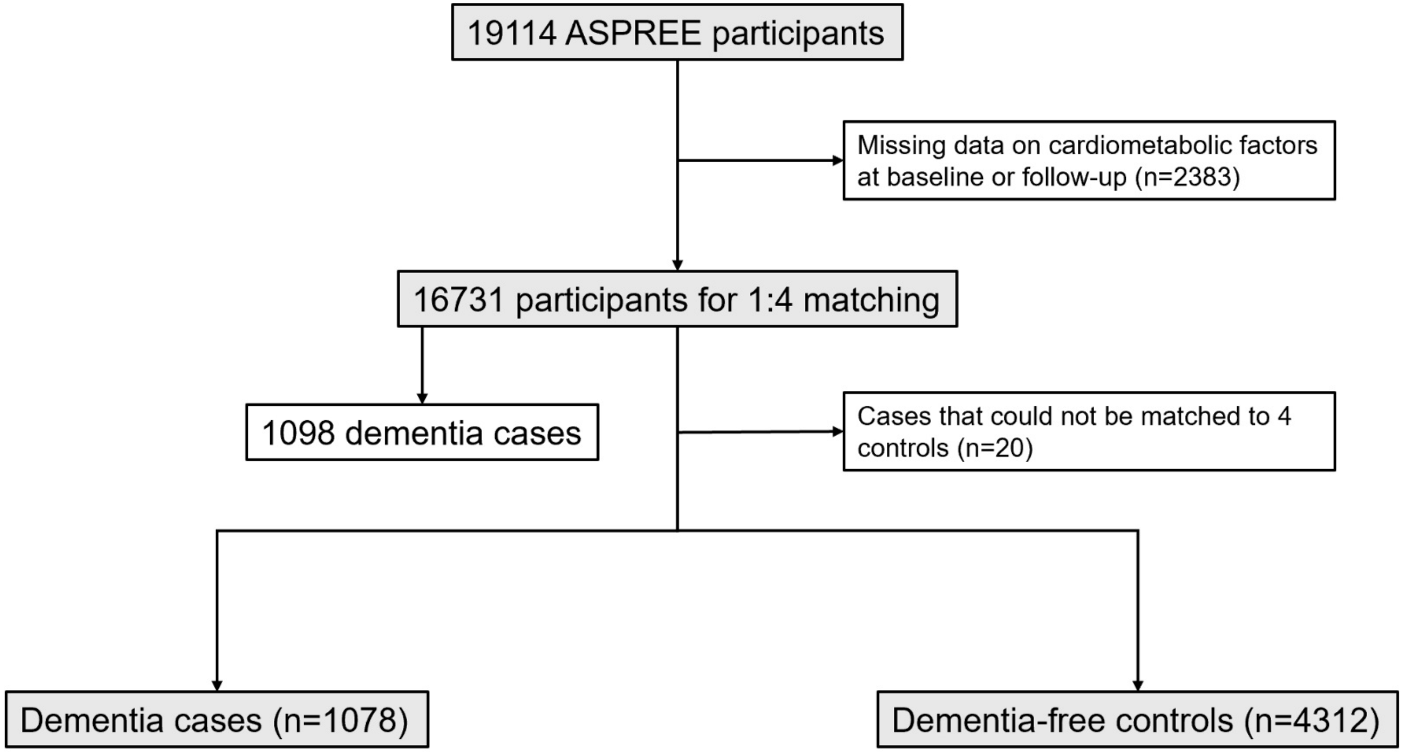

eFigure 1. Flow diagram of participant selection. Note: Cases and controls were matched by age at time 0, gender, race and ethnicity and years of education.

© 2025 Wu Z et al. *JAMA Network Open*.

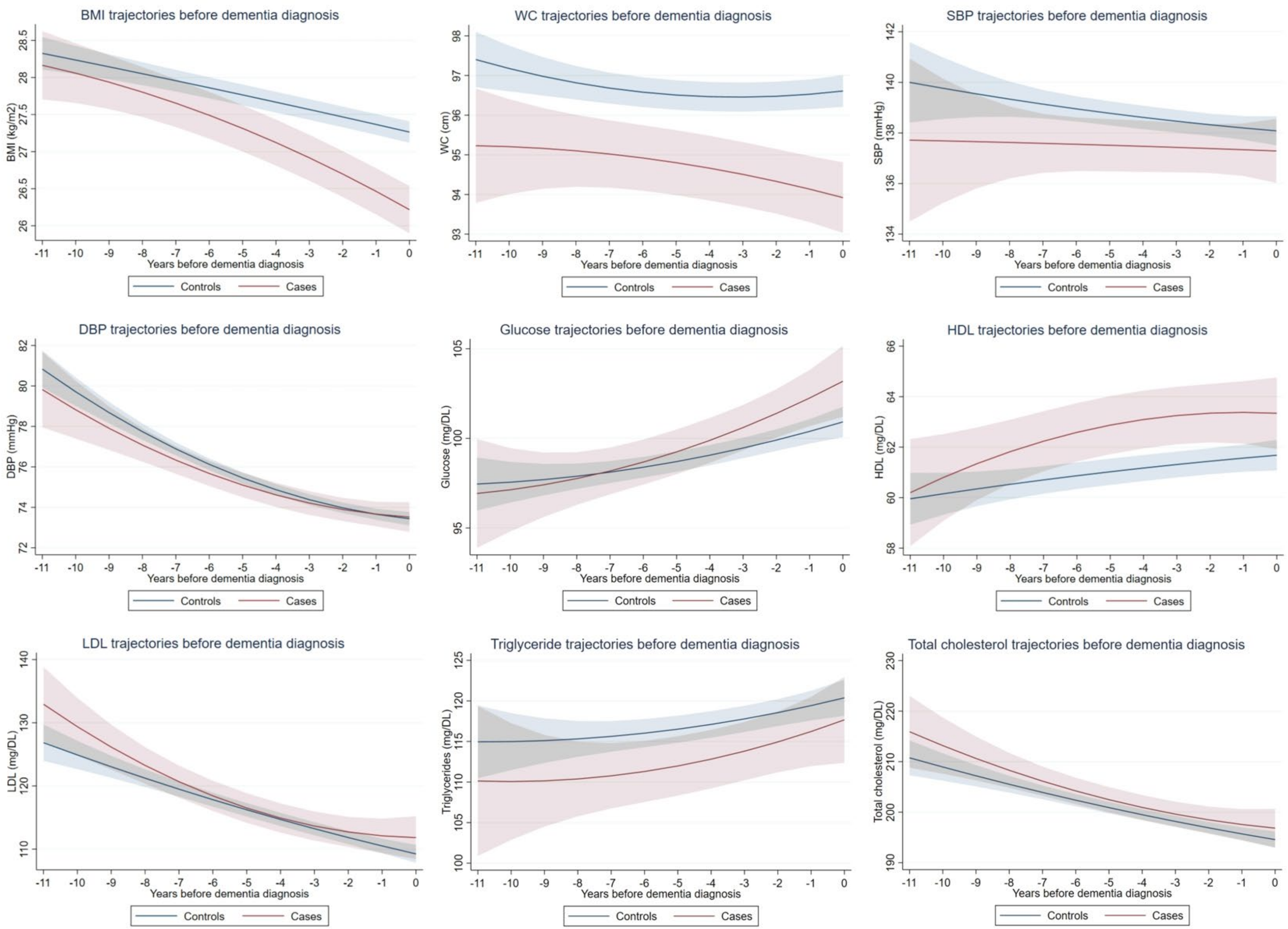

eFigure 2. Mean trajectories of cardiometabolic markers before dementia diagnosis by cases (n=749) and controls (n=3779), excluding participants who died during follow-up. Note: 1) The solid lines and shadings represent the predicted mean trajectories and the 95% confidence intervals of the corresponding cardiometabolic markers. 2) The models included case-control status, time, time^2, and their interaction, as well as age at time 0, gender, race and ethnicity and years of education.

© 2025 Wu Z et al. *JAMA Network Open*.

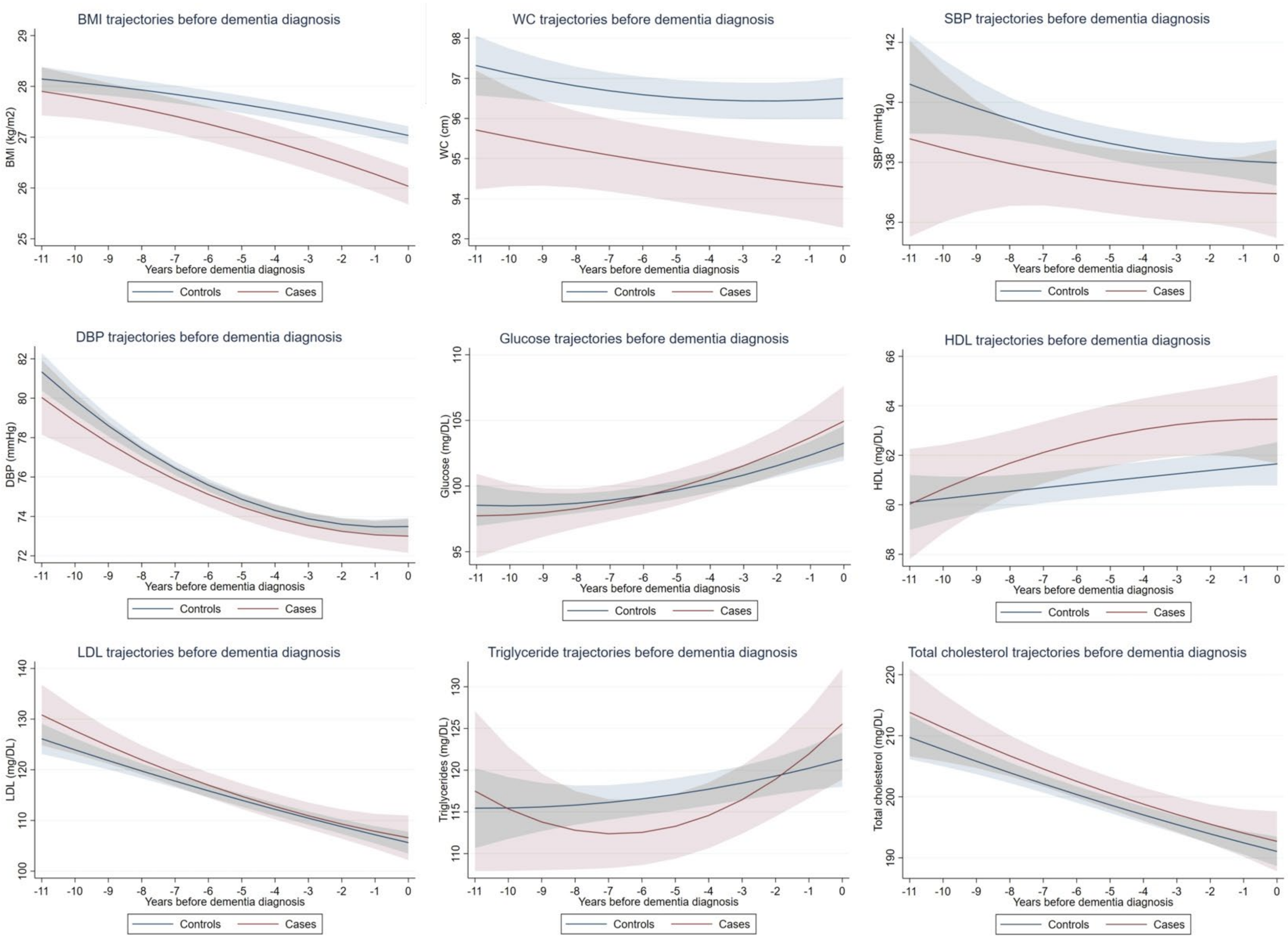

eFigure 3. Mean trajectories of cardiometabolic markers before dementia diagnosis by cases (n=593) and controls (n=2372), excluding participants matched within the first five years after enrollment. Note: 1) The solid lines and shadings represent the predicted mean trajectories and the 95% confidence intervals of the corresponding cardiometabolic markers. 2) The models included case-control status, time, time^2, and their interaction, as well as age at time 0, gender, race and ethnicity and years of education.

© 2025 Wu Z et al. *JAMA Network Open*.
