# Supplementary material for: Cardiometabolic Trajectories Preceding Dementia in Community-Dwelling Older Individuals
Source: JAMA Netw Open. 2025 Feb 7;8(2):e2458591. doi: 10.1001/jamanetworkopen.2024.58591 (PMC11806394; doi:10.1001/jamanetworkopen.2024.58591)
Supplement: Supplement 2. — Data Sharing Statement [file jamanetwopen-e2458591-s002.pdf]

Data Sharing Statement

Wu. Cardiometabolic Trajectories Preceding Dementia in Community-Dwelling Older Individuals. *JAMA Netw Open*. Published online February 7, 2025. doi:10.1001/jamanetworkopen.2024.58591

Data
Data available: No
